# A novel high-throughput activity assay for the *Trypanosoma brucei* editosome enzyme REL1 and other RNA ligases

**DOI:** 10.1093/nar/gkv938

**Published:** 2015-09-22

**Authors:** Stephan Zimmermann, Laurence Hall, Sean Riley, Jesper Sørensen, Rommie E. Amaro, Achim Schnaufer

**Affiliations:** 1 Institute of Immunology & Infection Research and Centre for Immunity, Infection & Evolution, University of Edinburgh, Edinburgh EH9 3FL, UK; 2 The Scripps Research Institute, 4122 Sorrento Valley Boulevard, San Diego, CA 92121, USA; 3 Department of Chemistry & Biochemistry and the National Biomedical Computation Resource, University of California, San Diego, CA 92093, USA

## Abstract

The protist parasite *Trypanosoma brucei* causes Human African trypanosomiasis (HAT), which threatens millions of people in sub-Saharan Africa. Without treatment the infection is almost always lethal. Current drugs for HAT are difficult to administer and have severe side effects. Together with increasing drug resistance this results in urgent need for new treatments. *T. brucei* and other trypanosomatid pathogens require a distinct form of post-transcriptional mRNA modification for mitochondrial gene expression. A multi-protein complex called the editosome cleaves mitochondrial mRNA, inserts or deletes uridine nucleotides at specific positions and re-ligates the mRNA. RNA editing ligase 1 (REL1) is essential for the re-ligation step and has no close homolog in the mammalian host, making it a promising target for drug discovery. However, traditional assays for RELs use radioactive substrates coupled with gel analysis and are not suitable for high-throughput screening of compound libraries. Here we describe a fluorescence-based REL activity assay. This assay is compatible with a 384-well microplate format and sensitive, satisfies statistical criteria for high-throughput methods and is readily adaptable for other polynucleotide ligases. We validated the assay by determining kinetic properties of REL1 and by identifying REL1 inhibitors in a library of small, pharmacologically active compounds.

## INTRODUCTION

Human African trypanosomiasis (HAT), also known as sleeping sickness, currently affects an estimated 30 000–50 000 people and threatens millions more (1,2). Despite a decreasing number of new infections over the last years, HAT still has a major impact on health and economy of sub-Saharan Africa. A similar decline in cases in the 1960s was followed by re-emergence due to a collapse in surveillance and control activities (3). Additionally to the direct threat to human health, *Trypanosoma* spp. have a heavy impact on local economies by infecting livestock like cattle or pigs. The occurrence of resistant strains in humans has been reported but their distribution and frequency are unclear (4). Together, these problems illustrate the urgent need for new medication. HAT itself progresses in two stages, with the parasite *Trypanosoma brucei* proliferating in the host's blood and lymph during the first phase, making the parasites relatively accessible for drugs. However, phase I usually only shows unspecific symptoms like headache or fever and, if untreated, the disease progresses to phase II. Here the parasite enters the central nervous system (3,5). This results in various symptoms like coordination disabilities, confusion and disturbances in sleep cycle which gave rise to the common name sleeping sickness. Left untreated HAT is almost always fatal (6).

*Trypanosoma brucei* is an extracellular parasite from the order *Kinetoplastida*, infecting humans as well as many mammals (7,8). One of the most remarkable characteristics of kinetoplastids is the unusual organization of their mitochondrial genome, the eponymous kinetoplast (9). Mitochondrial proteins are encoded by a few dozen presumably identical maxicircles of ∼23 kbp. In addition, the kinetoplast also contains thousands of minicircles, each ∼1 kbp in size, encoding so called guide RNAs (gRNAs). These 50–60 nt transcripts are essential for the expression of mitochondrial genes by RNA editing, directing the process by virtue of their complementarity to fully edited mRNA (10). Three distinct protein complexes of ∼20S, called core editosomes (or RNA editing core complexes), modify the pre-mRNA by insertion or deletion of uridine-nucleotides (11–14). However, auxiliary complexes are required for gRNA processing and binding, and the editing process is integrated into other RNA processing mechanism, resulting in a highly complex network of interactions (14,15). A single gRNA can code for several insertions or deletions, but in most cases editing involves sequential action of several overlapping gRNAs. During editing the pre-mRNA is cut by an endonuclease, uridine nucleosides are inserted by a terminal uridylyl transferase (TUTase) or removed by an ExoUase, and finally the edited mRNA strand is re-ligated by an RNA ligase. Investigation of function and composition of the editosomes has shown that they contain two different trimeric subcomplexes. The first subcomplex contains structural protein KREPA2 (a.k.a. MP63), ExoUase REX2 and RNA editing ligase 1 (REL1), the second subcomplex contains structural protein KREPA1 (a.k.a. MP81), TUTase RET2 and RNA editing ligase 2 (REL2) (13,16). The structural proteins directly interact with the enzymes and bind them to the complex (17). While REL1 function is essential for *T. brucei* survival, no negative effect has been observed after REL2 RNAi-mediated knockdown (18–21), and the precise functions of the two RELs in uridine insertion and deletion editing remain to be clarified (14,21,22).

The RELs belong to the superfamily of polynucleotide ligases which also includes DNA ligases and other RNA ligases. This relationship is strongly supported by sequence comparison and structural investigations (23,24). All polynucleotide ligases share five conserved motifs (I, III, IIIa, IV, V) in their catalytic domain which form the active site and binding pocket for the cofactor adenosine triphosphate (ATP) or NAD^+^ (25). Members of this family employ a three-step reaction mechanism. The first step is adenylylation of a 100% conserved lysine residue by use of the ATP or NAD^+^ in the binding pocket. The second step is the transfer of AMP onto the 5′ end of the donor (3′) substrate RNA or DNA to yield a high-energy 5′-5′ pyrophosphate intermediate. In the third step the ligation of donor and acceptor (5′) RNA or DNA takes place and AMP is released.

The essential nature of REL1, the absence of a close homolog in the host and the availability of a high resolution crystal structure of its N-terminal catalytic domain (26) make it a very interesting target for drug development (18). As an important step toward this goal we have now developed a novel activity assay for REL1 that uses fluorophore labeled substrate RNA and is compatible with high-throughput screening (HTS) methods for the identification of small molecule inhibitors. Traditional REL1 activity assays utilize the incorporation of radioactively labeled ATP into the ligase or the ligation of pre-cleaved radioactively labeled RNA; both assays require gel electrophoresis (27). This makes these assays relatively complicated, expensive, time consuming and not amenable for high-throughput methods. HTS-compatible, fluorescence based assays have been described for DNA ligases (28–31) and for full-round trypanosome RNA editing (32,33), but not for RNA ligases.

To test the potential of our assay we used it to screen the Library Of Pharmaceutically Active Compounds (LOPAC, Sigma-Aldrich) for REL1 inhibitors. We confirmed activity of five of the top six hits in individual assays and assessed the potential binding mode of one confirmed hit, the anti-trypanosomatid drug suramin, by modeling the interaction with REL1 and applying molecular dynamics simulations.

## MATERIALS AND METHODS

### Protein production and purification

*Trypanosoma brucei* REL1 (systematic TriTrypDB ID Tb927.9.4360; www.tritrypdb.org) was produced recombinantly in *Escherichia coli* BL21 (DE3) cells. The REL1 expression construct, a generous gift from Wim Hol, Junpeng Deng and Meiting Wu (University of Washington, Seattle), was generated as follows. The sequence encoding REL1 amino acids 51–469 (corresponding to the mature polypeptide without the mitochondrial targeting signal) was cloned into pET-21d (Novagen). Additionally the sequence for a 6xHis-tag followed by a linker and amino acids 56–176 from KREPA2 (systematic TriTrypDB ID Tb927.10.8210), corresponding to the region directly interacting with REL1 (17), was cloned into the same vector downstream of the REL1 coding sequence. Competent BL21 (DE3) cells were transformed with this polycistronic construct and grown at 37°C to an OD of ∼0.4. Expression was induced with 0.1–0.5 mM IPTG and performed at 20°C for 20 h. Cells were harvested by centrifugation. The cell pellet was resuspended in 20 mM Tris/HCl pH 8.0, 500 mM NaCl, 10% (v/v) glycerol, 2 mM DTT, 0.1 mM PMSF and 1 mM benzamidine, supplied with Roche EDTA-free protease inhibitor. Cells were lysed with a TS Series Benchtop cell disruptor (Constant Systems Ltd.) and the lysate centrifuged at 50 000 *g* for 1 h. The supernatant was passed through a 0.2 μm filter and applied to a HiTrap FF IMAC (GE Healthcare) charged with Ni^2+^. After a wash step with 40 mM imidazole, protein was eluted with a linear 40–500 mM imidazole gradient. Elution fractions were analyzed by sodium dodecyl sulphate-polyacrylamide gel electrophoresis (SDS-PAGE) and suitable fractions were pooled. Protein was then concentrated to 10 mg/ml, flash frozen in liquid nitrogen and stored at -80°C.

### Assay development and optimization

Labeled and unlabeled synthetic RNA oligonucleotides were produced by IBA GmbH Göttingen, Germany, using phosphoramidites from Thermo Fisher Scientific; all oligonucleotides were purified by reversed phase HPLC. RNA for fluorophore labeling was synthesized with amino modifier C6-U at the respective position, and N-hydroxysuccinimide (NHS) esters of the dyes, which form an amide bond with the reactive amine, were used for labeling. Dye-labeled oligonucleotides were purified with an additional HPLC step after labeling. To determine the ideal conditions for RNA ligation and optimal FRET signal readout, several assay variables like type of fluorophore, temperature, buffer, pH, additives and denaturing methods were systematically tested. Initial RNA annealing and ligation conditions were as follows. Per reaction, 1 μl each of 5′-RNA (5′-AAG /U-6FAM/AU GAG ACG UAG G-3′), 3′-RNA (5′-pAUU GGA G/U-Cy5/U AUA Gp-3′) and gRNA bridge (5′-CUA UAA CUC CAA UCC UAC GUC UCA UAC UUp-3′) (all 10 μM) were mixed with 1 μl 10× reaction buffer (250 mM KCl; 125 mM HEPES pH 7.9; 50 mM Mg(OAc)_2_; 2.5 mM DTT; 1% (w/v) Triton X-100) and 6 μl H_2_O, incubated at 70°C for 2 min and cooled to 20°C at 0.1°C/s. A total of 50 ng of REL1 were mixed in a total volume of 10 μl with 1 μg BSA, 1 μl 200 μM ATP and 1 μl 10× reaction buffer. Enzyme and substrate mix were combined and incubated at 27°C for 30 min. For heat denaturation, 80 μl stop buffer (25 μM competitor DNA oligonucleotide 5′-AAG TAT GAG ACG TAG GAT TGG AGT TAT AG-3′; 12.5 μM EDTA) was added, the sample incubated at 95°C for 2 min and then transferred to a 96-well plate (Greiner Bio-One 655083). Fluorescence was measured in a BMG Labtech FLUOstar Omega plate reader (excitation at 490 nm, detection at 670 nm).

### Assay conditions for HTS in 384-well format

Substrate RNAs were annealed in the presence of 25 mM KCl, 12.5 mM Tris–HCl pH 8.0, 5 mM Mg(OAc)_2_ and 0.25 mM DTT at a concentration of 1 μM in 10-ml batches by incubation at 70°C for 10 min, followed by slow cooling to room temperature. Annealed substrate was stored at −80°C. For 2400 assays, 2.4 μl 100 mM ATP were added to 12 ml pre-annealed substrate and 5 μl of the mixture dispensed into each well of a 384-well assay plate (Greiner 788075). A total of 50 nl of LOPAC (Sigma-Aldrich; 2 mM in DMSO) or Maybridge Hitfinder (Thermo Fisher Scientific; 2 mM in DMSO) compounds were added per well using a Biomek FX instrument. Recombinant REL1 was diluted in 10% (v/v) glycerol, 300 mM NaCl, 2 mM DTT and 20 mM Tris–HCl pH 6.0 to a final concentration of 300 ng/μl. One hundred twenty microliter REL1 solution were added to 11.88 ml ligation buffer (5 mM KCl; 12.5 mM Tris–HCl pH 6.0; 1 mM Mg(OAc)_2_; 0.25 mM DTT, 0.2% (w/v) Triton X-100; 0.003% (w/v) Brij 35) and 5 μl of the REL1/ligation buffer mix were added to each well to start the reaction (15 ng/μl REL1 per well). A total of 50 μM NSC-42067 served as positive control for inhibition (34). As additional controls, each plate included reactions without inhibitor and reactions without REL1. After 30 min incubation at room temperature the reaction was stopped by adding 12.5 μl STOP solution (9 M urea; 20 mM EDTA). After a minimum incubation period of 15 min plates were read using a ratiometric wavelength read (excitation at 485 nm, detection at 665 and 535 nm). HTS was carried out at The Scripps Research Institute, San Diego, CA, USA.

### Gel electrophoresis

After ligation reaction, 20% (w/v) polyacrylamide gels were used to separate the RNA molecules by non-denaturing PAGE in Tris-borate-EDTA (TBE) buffer. For denaturing PAGE, gels contained 7 M urea. Gels were analyzed using a Typhoon FLA 7000 (GE Healthcare).

### Protein docking and modeling

The first model of suramin bound simultaneously to the active sites of two REL1 enzymes was built manually using Maestro from Schrödinger (35). Initially, half of suramin, which is symmetrical, was modeled into the active site of the REL1 crystal structure (26). Structural water molecules were deleted prior to this step to allow positioning of the ligand. The resulting complex was minimized to optimize enzyme-ligand interactions. Subsequently, a copy was made of that complex, which was then reflected in the symmetry point of suramin. The two halves of suramin were subsequently bonded. To generate subsequent models, single bonds in suramin were rotated while the enzymes with respect to the ligand were fixed; this forced a rigid body rotation of the enzymes with respect to one another to find allowed conformations where the enzymes did not clash. To evaluate the stability of both the ligand binding mode and the proteins adjacent to each other, molecular dynamics simulations of the complexes were carried out (further details can be found in the Supplementary Data).

## RESULTS

### Protein production and purification

**Figure 1.**
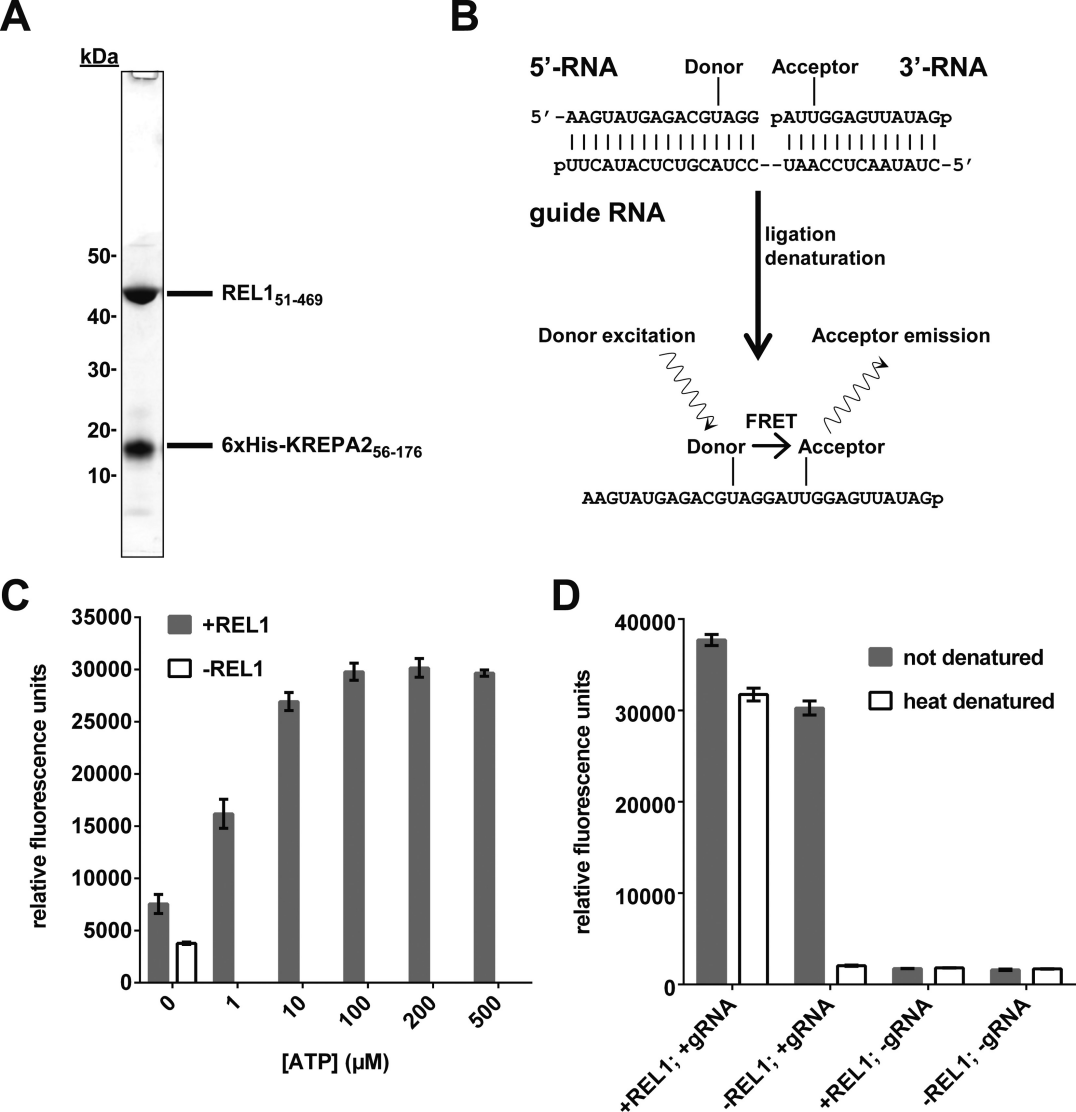
Purification of recombinant REL1 and initial characterization of the FRET-based assay. (**A**) SDS-PAGE showing the purified REL1_51–469_-KREPA2_51–176_ complex. REL1_51–469_ has a size of 46.9 kDa, while 6xHis-KREPA2_51–176_ has a size of 16.5 kDa. (**B**) Schematic representation of the assay principle. A nicked RNA duplex consisting of guide RNA (gRNA), 5′-RNA with donor fluorophore and 3′-RNA with acceptor fluorophore serves as substrate. After incubation with REL1, ligated RNA is detected by denaturation-resistant FRET between the two fluorophores. (**C**) The FRET signal is dependent on ATP and REL1. The assay was carried out as described in ‘Materials and Methods’ section under ‘Initial RNA annealing and ligation conditions’, except 100 ng REL1 and the concentrations of ATP indicated were used. Means and standard deviations for three independent experiments are shown. (**D**) The FRET signal is dependent on gRNA. The assay was carried out as described in ‘Materials and Methods’ section under ‘Initial RNA annealing and ligation conditions’, except 200 ng REL1 were used, gRNA was added or omitted as indicated and samples were or were not denatured at 96°C before reading, as indicated. Means and standard deviations for five (+gRNA) or three (−gRNA) replicates are shown.

We were unable to obtain soluble, full-length REL1 when expressed on its own in *E. coli*. However, a di-cistronic construct for co-expression of REL1 with a fragment of KREPA2 (amino acid residues 56–176; plasmid generously provided by Wim Hol, University of Washington), permitted expression of soluble REL1. This region of KREPA2 interacts with the C-terminal domain of the ligase (17,36) and probably supports its correct folding in *E. coli*. Recombinant protein expression in *E. coli* BL21 (DE3) using LB medium resulted in an initial ratio of roughly 2:1 of insoluble to soluble protein (estimated from relative band intensity on Coomassie-stained SDS-PAGE gels). Reducing IPTG concentration from 0.5 mM to 0.1 mM and adding a 10 min heat shock at 42°C directly before induction resulted in an optimized ratio of ∼1:1 and an increased yield of soluble REL1-KREPA2_56–176_ protein complex. Using a 6xHis-tag at the N-terminus of KREPA2_56–176_ for IMAC purification, the REL1-KREPA2_56–176_ complex could be isolated to >95% purity (Figure 1A).

### Assay development and optimization

Synthetic substrates developed for traditional *in vitro* editing and ligation assays (37) were modified by internal attachment of the donor (6-FAM) and acceptor fluorophore (Cy5) of a Förster Resonance Energy Transfer (FRET) pair to the 5′-RNA and 3′-RNA oligonucleotides, respectively. After annealing of 5′-RNA and 3′-RNA to a complementary gRNA, close proximity of the two fluorophores in the dsRNA results in FRET. After sealing of the nick (e.g. by active REL1), the FRET signal will be unaffected by dsRNA denaturation (Figure 1B). Preventing sealing of the nick (e.g. by inhibition of REL1 activity) will result in loss of the FRET signal after dsRNA denaturation. As shown in Figure 1C and D, the FRET signal shows the expected dependency on REL1, ATP and gRNA. Reactions without exogenous ATP gave a slightly higher signal than reactions without REL1 (Figure 1C). This has previously been observed for editosomes purified from *T. brucei* (37,38) as well as for recombinant phage RNA ligase T4Rnl2 (24,39)—the closest known homolog of the RELs (26)—and is due to some ligase being pre-adenylylated when isolated from cells. The dependence on gRNA is consistent with earlier reports that unbridged RNA molecules are poor substrates for the RELs (37,38). Efficient denaturation was achieved by incubating the samples at 96°C in the presence of 200-fold excess of a DNA oligo that is complimentary to the gRNA. This reduced the signal of unligated RNAs to that of control reactions without both gRNA and REL1 (Figure 1D, compare heat denatured ‘−REL, +gRNA’ to ‘−REL1, −gRNA’). Gel electrophoresis confirmed generation of the expected products after annealing of the synthetic RNA substrates and incubation with REL1 (Supplementary Figures S1 and S2). For both fluorophores, two alternative attachment sites were also tested (6-FAM attached to the first uridylyl on the 5′-RNA and Cy5 attached to the third uridylyl on the 3′-RNA, compare to Figure 1B; the attachment chemistry limited the possible attachment sites to uridylyls). The RNA molecules were tested in all four combinations. The combination shown in Figure 1B gave an at least three-fold better signal-to-baseline ratio (S:B; baseline defined as reaction without ligase) than the other combinations (data not shown) and was used for further optimizations. Replacing 6-FAM with Cy3 as donor resulted in an approximately five-fold drop in S:B (Supplementary Figure S3A and B). Consequently, subsequent optimizations were performed using the 6-FAM/Cy5 combination (excitation at 490 nm, detection at 670 nm). Note that the binding kinetics for unlabeled or fluorophore-labeled RNA substrates to REL1 were not determined. It is possible that the fluorophores affect binding to REL1, for example by steric hindrance, and such effects may in part be responsible for the differences in S:B ratios observed for the various combinations tested above.

**Figure 2.**
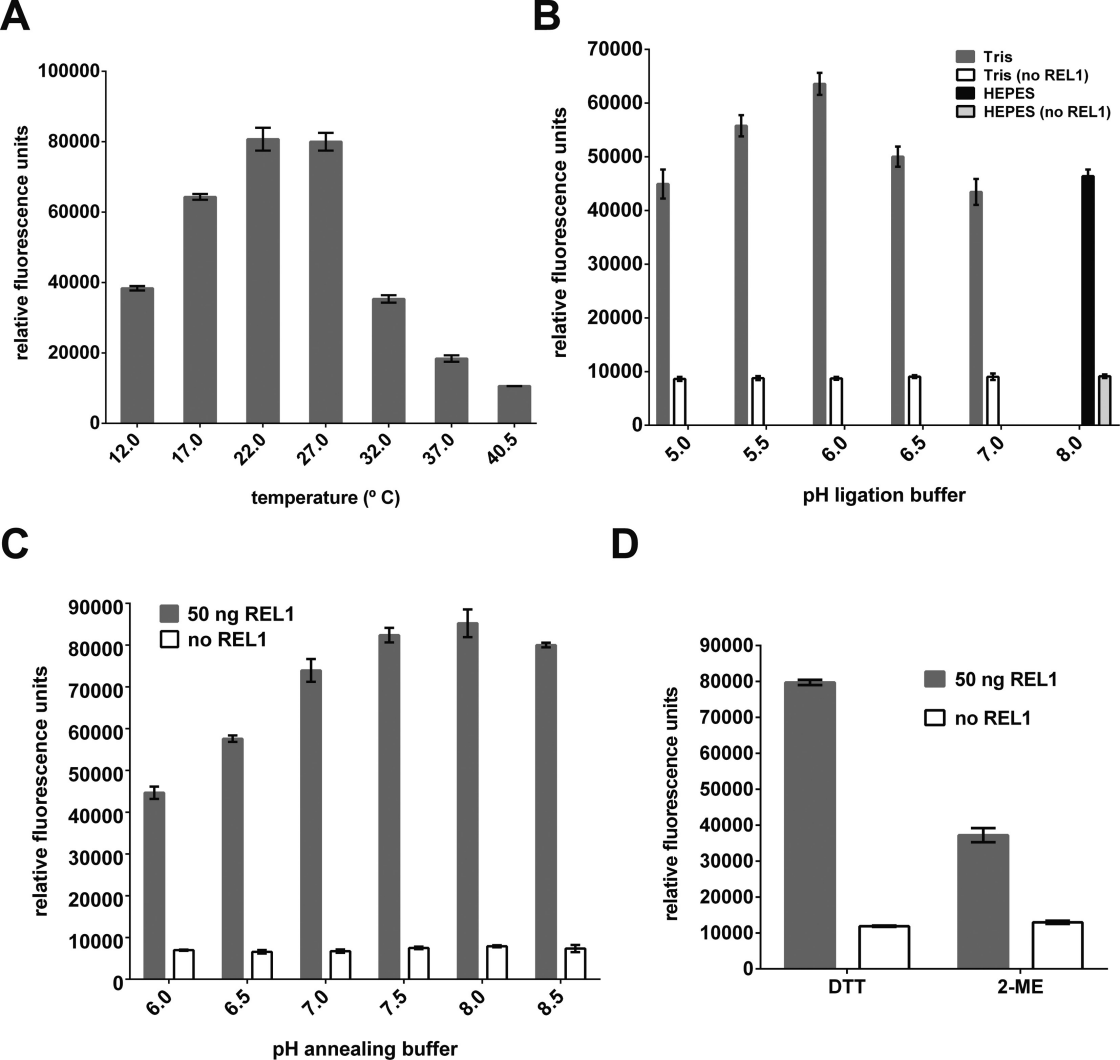
Optimization of assay conditions (I). Optimization of (**A**) incubation temperature, (**B**) pH of ligation buffer, (**C**) pH of annealing buffer. (**D**) Assay performance with dithiothreitol (DTT) versus β-mercaptoethanol (2-ME) as reducing agent. For (B), (C) and (D), parallel assays without REL1 were carried out as controls. Except for the parameters tested, all experiments were carried out as described in ‘Materials and Methods’ section under ‘Initial RNA annealing and ligation conditions’, and means and standard deviations for three replicates are shown.

To determine suitable temperatures for the ligation reaction a range from 12.0–40.5°C was tested. As shown in Figure 2A, 22°C and 27°C resulted in the strongest FRET signals, with higher temperatures possibly decreasing enzyme and/or substrate stability. Testing a pH range of HEPES and Tris–HCl buffers for the ligation reaction showed that Tris–HCl pH 6.0 gave the best S:B ratio (Supplementary Figure S3C, Figure 2B). Similar experiments using different buffers and pH values for the RNA annealing reaction showed that Tris–HCl buffer with pH 8.0 resulted in the best S:B ratio (Figure 2C; note that the resulting pH in the ligation reaction is 7.0). Several additional buffer components were tested to enhance ligation efficiency. Of the Mg^2+^ and K^+^ concentrations tested, 50 mM Mg^2+^ and 250 mM K^+^ in the 10× annealing buffer and 10 mM Mg^2+^ and 50 mM K^+^ in the 10× ligation buffer were optimal (data not shown). The presence of six cysteines in the REL1–KREPA2_56–176_ complex, four in REL1 and two in KREPA2_56–176_, makes formation of disulphide bonds a possible way of protein denaturation. Accordingly, 2.5 mM DTT was used during purification and in all activity assays. However, since 2-mercaptoethanol (2-ME) has a longer half-life than DTT (40) it was tested as an alternative reducing agent. Initial experiments with pre-ligated FRET substrate indicated that 2-ME had no negative effect on the FRET signal (data not shown). However, independently of buffer or pH, 0.2% (v/v) 2-ME resulted in a much lower S:B ratio after the ligation reaction compared to 2.5 mM DTT (Figure 2D). It is possible that the smaller 2-ME can enter the REL1 ATP binding pocket and modify a cysteine involved in forming the active center (26), thereby altering the affinity and activity of REL1. The larger DTT would not be able to enter the binding pocket.

**Figure 3.**
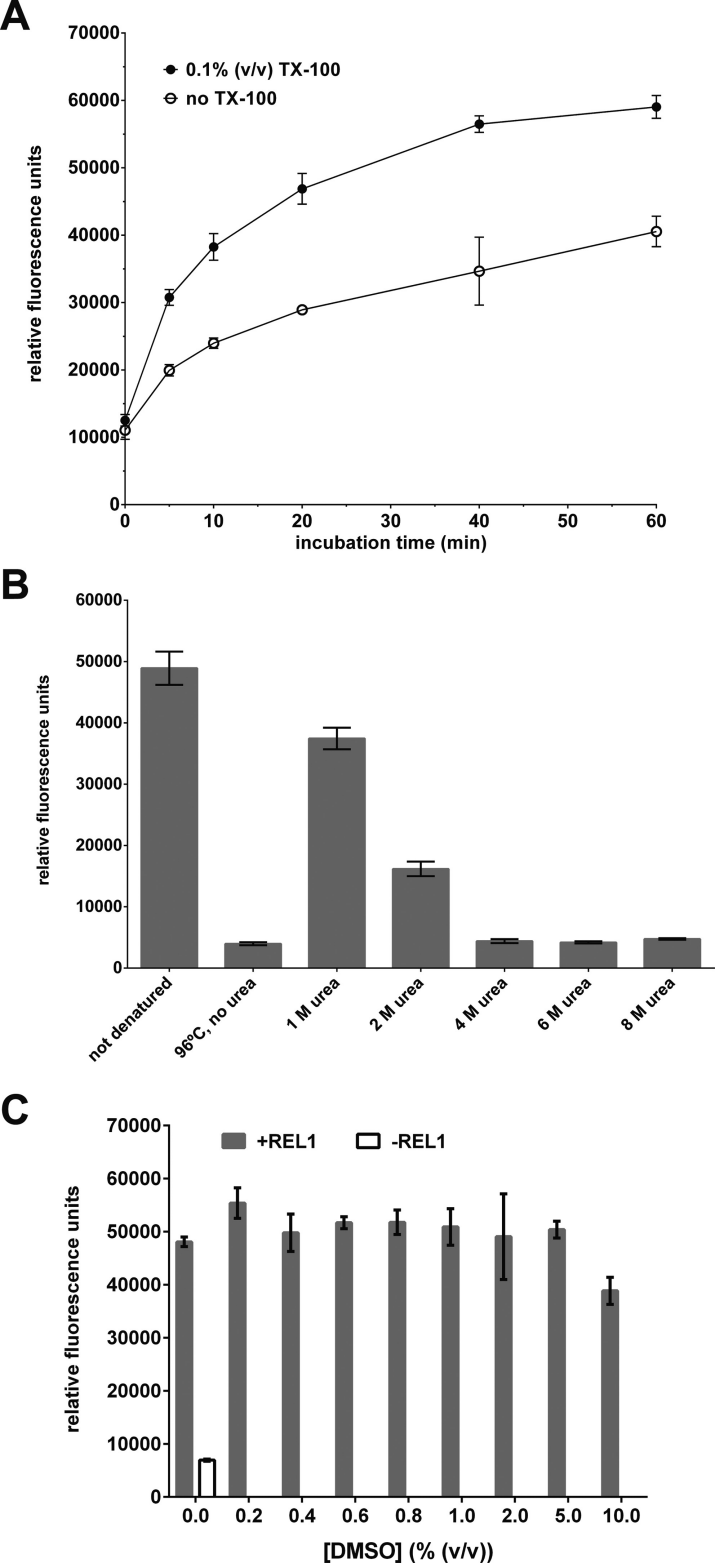
Optimization of assay conditions (II). (**A**) Comparison of ligation production formation over time in the presence and absence of 0.1% (v/v) Triton X-100 (TX-100). (**B**) Comparison of different methods for disrupting the annealed (but unligated) nicked dsRNA substrate. Denaturation by 96°C heat in the presence of 200-fold excess competitor DNA oligo was compared to treatment with 1–8 M urea at room temperature. (**C**) Influence of 0.2–10% (v/v) DMSO on assay performance. Except for the parameters tested, all experiments were carried out as described in ‘Materials and Methods’ section under ‘Initial RNA annealing and ligation conditions’, and means and standard deviations for three replicates are shown.

The presence of detergents in the assay proved beneficial. A final concentration of 0.1% (w/v) Triton X-100 improved the S:B ratio and resulted in faster reaction kinetics (Figure 3A). Titration of Triton X-100 and Brij-35 showed that a combination of 0.1% (w/v) Triton X-100 and 0.003% (w/v) Brij-35 gave the best results (data not shown). The small amount of detergents might stabilize REL1 or KREPA2_56–176_ by increasing protein solubility, reduce protein binding to the well surface and/or help release ligated RNA product from REL1.

In our assay, distinguishing ligated from un-ligated substrate with good sensitivity and specificity depends upon a complete separation of the fluorophore-labeled RNAs from the gRNA bridge at the end of the reaction. Heat denaturation at 96°C in the presence of excess competitor DNA (complementary to gRNA) proved efficient in small scale experiments (see Figure 1D). However, this method is not ideal for an HTS setting. Therefore, urea concentrations between 1 M and 8 M were tested as a method for efficient denaturation at room temperature. Concentrations of 4 M urea or higher denatured RNA duplexes as effectively as incubation at 96°C (Figure 3B). The assay was not affected by the presence of up to 5% (v/v) DMSO (Figure 3C), an important condition for HTS where compounds are usually available in this solvent. Only concentrations ≥ 10% (v/v) DMSO resulted in a noticeable decrease of the FRET signal.

### Characterization of REL1 reaction kinetics using the FRET assay

**Figure 4.**
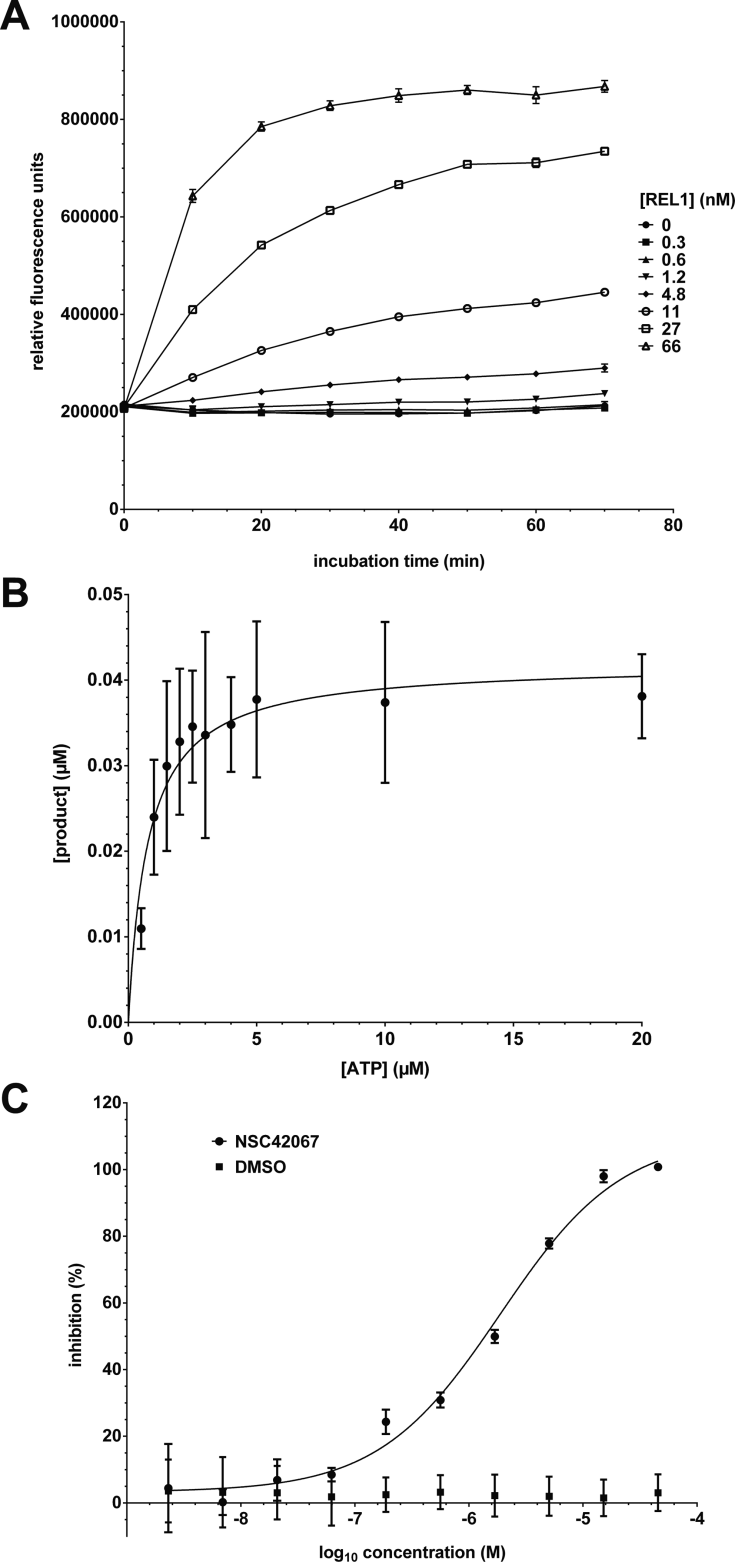
Kinetic characterization of REL1. (**A**) Product formation over time. Substrate was incubated with different concentrations of REL1 and urea stop buffer was added after 0–70 min. (**B**) Determination of K_m_ for ATP. The assay was carried out with varying concentrations of ATP and 50 ng REL1 in a reaction volume of 20 μl. The data were fitted to the Michaelis–Menten equation. (**C**) Determination of IC_50_ for compound NSC42067. REL1 activity was measured in the presence of increasing concentrations of compound, calculated as% inhibition compared to the control, plotted and fitted to a sigmoidal curve. All experiments were carried out under optimized conditions, and means and standard deviations for three replicates are shown.

Using the optimized conditions we next measured product formation over time and over a range of REL1 concentrations (Figure 4A). As expected, product formation increased with higher protein concentration as well as longer incubation times. For 66 nM REL1, the maximum signal was reached after 20 min. Calibration with a double 6-FAM/Cy5-labeled control oligonucleotide determined that the maximum signal corresponded to ligation of ∼95% of substrate (Supplementary Figure S3D). Reactions with lower REL1 concentrations did not reach the same maximum. Adding fresh ATP restarted the reaction (Supplementary Figure S3E), suggesting that this observation was not simply a consequence of deteriorating activity of the enzyme. The biochemical basis for this observation remains to be investigated. Testing product formation under the assumption of Michaelis–Menten kinetics using a range of ATP concentrations and initial velocity conditions (up to 5 min) established K_m_, V_max_ and k_cat_ values for the co-substrate ATP of 0.76 ± 0.27 μM, 42.0 ± 4.0 nM/min and 0.54 ± 0.04 min^−1^, respectively (Figure 4B). The specific activity of the REL1 preparation under these reaction conditions was therefore 16.8 nmol min^−1^ mg^−1^ (50 ng of protein was used per assay volume of 20 μl); this value was similar for other preparations. We caution that the kinetic parameters were calculated under the assumption that 100% of the enzyme in the preparation was active. The actual percentage of active protein has not been determined.

### Pilot HTS and identification of REL1 inhibitors

**Figure 5.**
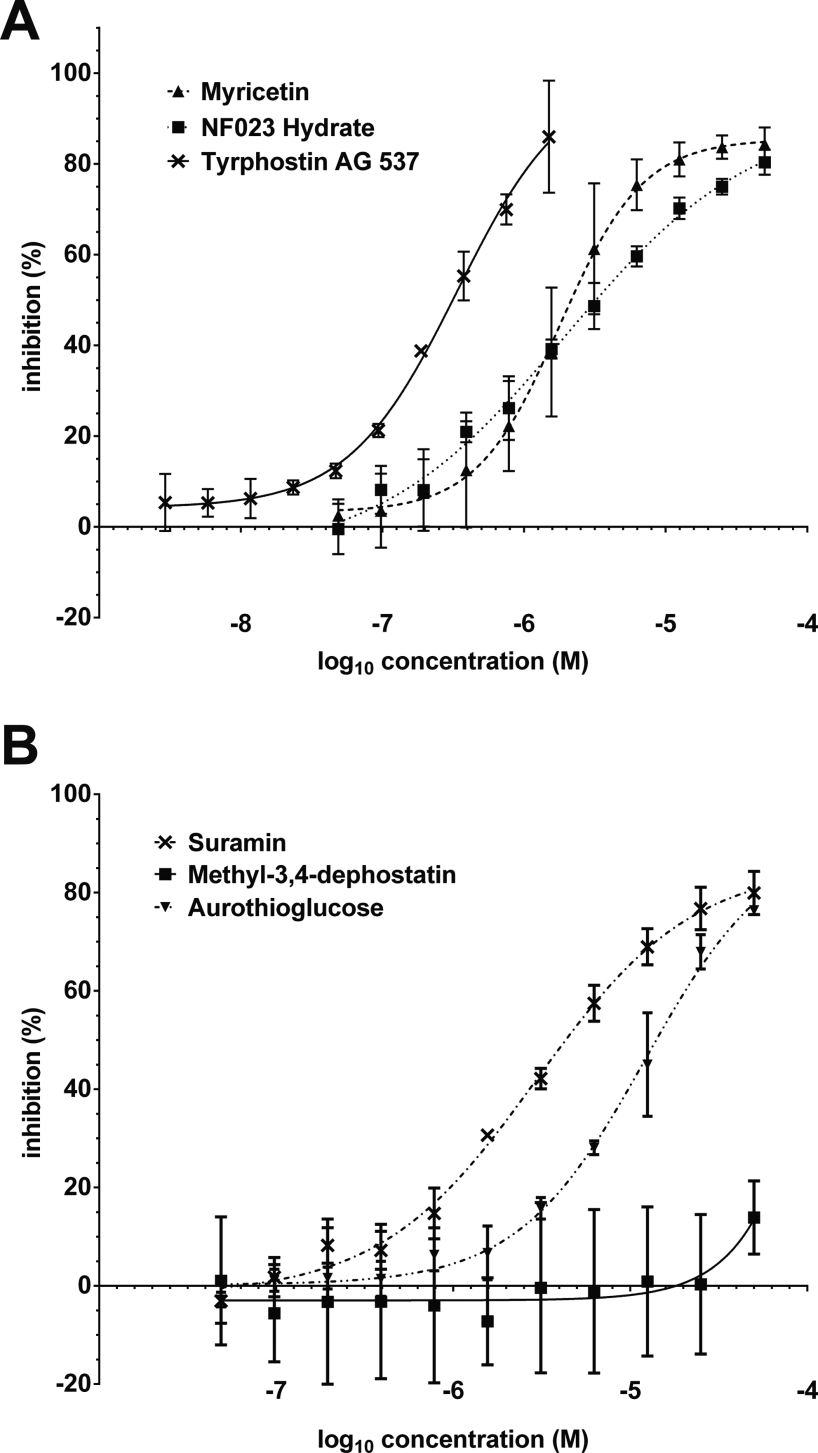
Dose-response curves for the top six hits from the pilot screen against the LOPAC library. Data were plotted as% inhibition compared to controls without inhibitor and fitted to a sigmoidal curve. (**A**) Compounds myricetin, NF023 hydrate and tyrphostin AG537. (**B**) Compounds suramin, methyl-3,4-dephostatin and aurothioglucose. All experiments were carried out under optimized conditions, and means and standard deviations for three replicates are shown.

To validate the suitability of the FRET-based REL1 activity assay for HTS, the assay conditions were adapted to 384-well format to facilitate a pilot screen. An ATP concentration of 10 μM was chosen as a compromise between sensitivity to inhibition and robust S:B ratio. As positive control for inhibition we used National Cancer Institute compound NSC42067, which had previously been identified in a virtual screen and experimentally confirmed as a REL1 inhibitor that interferes with the first enzymatic step, adenylylation (34). Using the HTS assay conditions an IC_50_ of 1.4 μM was determined for NSC42067 (Figure 4C; see also Supplementary Figure S4). Screening the LOPAC 1280 library (single shot assays at 10 μM final concentration; four 384-well plates, including controls) resulted in a mean Z’ score of 0.74 and a mean S:B of 2.3, confirming suitability for HTS (41). Applying the standard hit cut-off of three standard deviations from control samples resulted in the identification of 30 initial ‘hits’ (2.4% hit rate). Eight of these were not reproducible, resulting in a final hit list of 22 compounds (Table 1). Six of the seven compounds that showed the strongest inhibition in the single-shot screen (Supplementary Figure S5) were analyzed by dose response curves to determine IC_50_ values. The compounds myricetin, tyrphostin AG 537, suramin, NF 023 hydrate and aurothioglucose could be confirmed as inhibitors with IC_50_ values in the low μM range, while the initial hit methyl-3,4-dephostatin showed no significant inhibition up to 50 μM concentration (Figure 5 and Table 1). A second pilot screen against the Maybridge library with a slightly altered liquid handling protocol (pre-dispensing 25 μl of enzyme dilution to each of the first two well of the plate) resulted in improved HTS statistics of an average Z’ of 0.88 and an S:B of 3.6 (Supplementary Figure S6). The standard cut-off of three standard deviations from control samples resulted in a 3.3% hit rate. An additional reading before stopping the reaction allowed elimination of compounds interfering with the FRET signal and reduced the hit rate to <1%.

**Table 1.** Hits from the pilot screen against the LOPAC library

Name	CAS No.	EC No.	PubChem SID	Molecular weight	Inhibition at 10 μM^1^	IC_50_ (μM)^2^
Myricetin	529-44-2	208-463-2	24278566	318.24	100%	1.78
Methyl-3,4-dephostatin	n/a	n/a	24278575	168.15	97%	> 50
Tyrphostin AG 537	n/a	n/a	24278741	448.43	93%	0.25
Aurothioglucose hydrate	12192-57-3	235-365-7	24724383	392.18	93%	12.14
NF 023 hydrate	104869-31-0	n/a	24278597	1162.88	79%	1.95
Iofetamine hydrochloride	95896-48-3	n/a	24277716	339.64	70%	n/d
Suramin sodium salt	129-46-4	204-949-3	24277738	1429.17	66%	2.94
β-Lapachone	4707-32-8	n/a	24278512	242.27	48%	n/d
Cefsulodin sodium salt hydrate	52152-93-9	257-692-4	24278340	554.53	46%	n/d
L-Canavanine	543-38-4	n/a	24892427	176.17	46%	n/d
R-(–)-Desmethyldeprenyl hydrochloride	115586-38-4	n/a	24278383	209.72	38%	n/d
cDPCP	106343-54-8	n/a	n/a	379.14	30%	n/d
ZM 39923 hydrochloride	n/a	n/a	24278044	367.91	26%	n/d
Auranofin	n/a	251-801-9	n/a	678.48	24%	n/d
8-(p-Sulfophenyl)theophylline hydrate	80206-91-3	n/a	24277683	336.32	23%	n/d
MNS	1485-00-3	n/a	n/a	193.16	22%	n/d
2-(Methylthio)adenosine 5′-diphosphate trisodium salt hydrate	475193-31-8	n/a	24278816	539.24	22%	n/d
Cephalosporin C zinc salt	59143-60-1	261-624-9	24278307	478.79	21%	n/d
2-Chloroadenosine triphosphate tetrasodium hydrate	n/a	n/a	24277700	629.55	18%	n/d
MDL 105,519	161230-88-2	n/a	24277992	376.19	18%	n/d
Morin hydrate	654055-01-3	207-542-9	24278552	302.24	17%	n/d
cis-Diammineplatinum(II) dichloride	15663-27-1	239-733-8	24278632	300.05	14%	n/d

^1^As suggested by HTS; n/a, not available; n/d, not determined.

^2^Note that the IC_50_ values measured will be influenced by the percentage of active protein in the preparation, which in this case is not known.

### Modeling suramin into the REL1 active site

**Figure 6.**
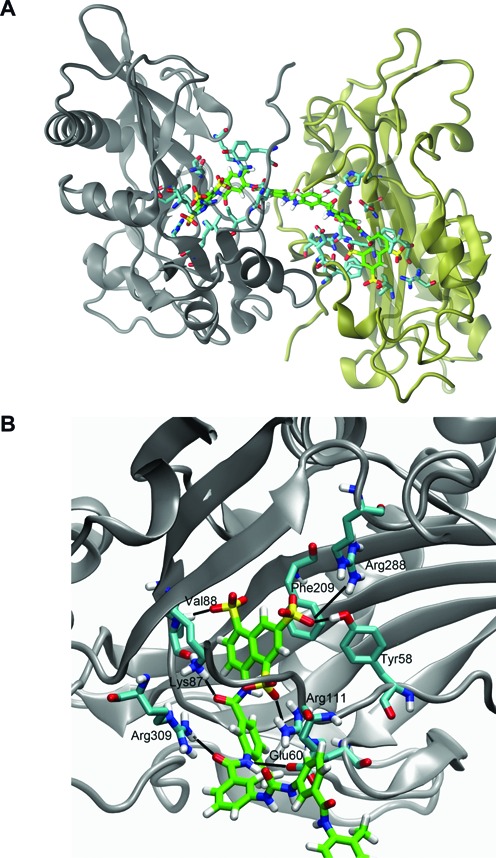
Proposed binding mode of suramin to REL1. (**A**) Predicted binding of suramin to two REL1 enzymes simultaneously. The two enzymes have been colored gray and tan, respectively. Suramin is shown with its carbon atoms colored green. Only side-chains of residues in the two enzymes that interact with suramin are shown for clarity (carbon atoms colored cyan). (**B**) Detailed view of the predicted interaction of one half of suramin with key residues in the ATP binding pocket. Suramin, shown with carbon atoms colored green, assumes the binding position of REL1's natural substrate ATP and its interactions with the enzyme. Only the side-chains of interacting residues in the enzyme are shown for clarity (in cyan).

Suramin's relatively high potency combined with its large size and symmetric character suggested that it might be able to bind two instances of REL1 simultaneously. Indeed, simultaneous binding of this compound to two target molecules has been shown for *Leishmania mexicana* pyruvate kinase (*Lm*PYK) (42). Therefore we explored whether models of suramin binding in the ATP binding pocket of two REL1 enzymes could be built without the two enzymes clashing. To create the initial model of such a complex we took advantage of the symmetry in suramin and first modeled binding of the one half to a single REL1 enzyme using the crystal structure with ATP bound (26). Half of suramin was placed in the active site of REL1 overlaying the naphthalene moiety of suramin with that of ATP as best possible—given the bulky sulfonate groups—while avoiding steric clashes. The complex was then duplicated and reflected in the symmetry point of suramin shown in Figure 6A. The binding mode of suramin is very similar to other sulfonated naphthalene inhibitors, V2–V4, that we have previously discovered (34) and the naphthalene ring's position is very similar to that of the native ATP substrate, also stacking with Phe209 (Figure 6B; see Supplementary Data for further details of the binding mode). Differences are likely due to suramin containing three bulky sulfonate groups while the other naphthalenes contained only one or two of these. Exploring different orientations of the two enzyme molecules by rigid-body rotation we found three additional models, during the rotation where the enzymes did not clash. To gauge whether the four models were stable, we subjected each of them to 100 ns of molecular dynamics (MD) simulations. The enzymes are independently very stable over the course of the simulations (Supplementary Figure S7), the protein–ligand interactions are stable, and the water phase between the two enzymes is persistent throughout each of the simulations. This latter part suggests that the two enzymes do not collapse on each other as a result of suramin binding, which is in part perhaps because the length of suramin prevents the two proteins from clashing. The salt-bridge between Glu60 and Arg111, which was previously reported to be dynamic during simulations (34,43), shows similar behavior here; the interaction is broken and intermittently formed during the simulation of some of the models, in other models it is more stable, perhaps attributed to Glu60's interacting with an amine group of suramin. The models suggest that suramin binding to two proteins is a likely outcome and contributes to its relatively high potency. The binding mode we modeled for suramin is also likely for NF 023 hydrate, since these molecules are very similar, with NF 023 hydrate having a shorter linker in the middle, allowing the two enzymes to come significantly closer together.

## DISCUSSION

The key editing enzyme REL1 has been validated as an essential enzyme in the disease-causing bloodstream form of *T. brucei* (18), but lack of a specific, HTS compatible activity assay has hampered development of REL1 as a target for novel therapies. We have now developed a novel FRET-based assay for REL1 that combines ease of use with highly reliable and reproducible results, thus demonstrating its suitability for HTS. Together with available HTS assays that can monitor a complete cycle of editing *in vitro* (32,33), this assay makes the unique RNA editing process in trypanosomatid parasites accessible as a new target for therapeutic intervention.

Several aspects of this assay have systematically been optimized for the use in drug discovery. For example, the use of urea instead of heat for denaturation reduces handling time and eliminates the need for a suitable method of heating. One major advantage of the FRET-based assay is the direct measurement of the ligated RNA product itself. Avoiding indirect measurement through secondary products eliminates possible sources for errors. Using only labeled RNA substrate enables detection of every ligated RNA strand, dramatically increasing the S:B ratio. Given enough time and sufficient protein concentration, almost all of the supplied substrate is ligated, which increases the S:B ratio further. Combined, these aspects result in robust screening statistics and biologically meaningful hit identification: five of the six hits that were selected for follow-up could be confirmed. The absence of sample heating, the easy execution due to the low number of steps and components and the low volume needed compare favorably to assays described for DNA ligases (28–31).

Although the principal motivation for screening the LOPAC library was to validate our assay for HTS purposes, the hits that were confirmed as REL1 inhibitors (suramin, NF 023, myricetin, tyrphostin AG 537, aurothioglucose) may prove useful. Suramin has been used for treatment of HAT since the 1920s. Most likely it acts on several *T. brucei* pathways and the exact mode of action is still not entirely clear (42,44,45). Suramin and its analogue NF 023 have previously been identified as inhibitor of RNA editing *in vitro* (32,46). One study suggested that NF 023 and suramin ‘are either acting at a step following the endonuclease cleavage or alternatively having global effects on the editing complex’ (32). Our confirmation of these compounds as REL1 inhibitors supports the first hypothesis. Interestingly, our labs had previously identified related naphthalene-based compounds similar to suramin and NF 023 as REL1 inhibitors via virtual screens (34,43). However, since trypanosomes that are not dependent on mitochondrial gene expression show no decreased sensitivity to suramin (47), REL1 is clearly not a relevant target of suramin *in vivo*. Furthermore, negatively charged compounds would require active transport to reach suitable concentrations in the mitochondrial matrix, which is also negatively charged. Both suramin and NF 023 have also been found to inhibit HCV helicase, another ATP-dependent nucleic acid-modifying enzyme (48). Crystal structures of suramin and suramin analogues bound to pyruvate kinases from *L. mexicana* and *Trypanosoma cruzi* showed how suramin binds these proteins (42). Our molecular modeling studies suggest a similar two-to-one stoichiometry for REL1-suramin binding, and perhaps for NF 023 as well.

Two other hits from the LOPAC screen, myricetin and tyrphostin AG 537, share as a common feature phenyl groups with two or one additional hydroxyl groups, respectively, and this similarity could be a possible reason for the considerable potency of both molecules toward REL1. Nonetheless, they belong to different compound classes. Tyrphostin AG 537, like other dimeric tyrphostins, is a potent inhibitor of the oncoprotein EGF receptor tyrosine kinase (49) and of dynamin I GTPase activity (50). Its relatively high molecular weight makes it an unlikely candidate for hit-to-lead optimization. Myricetin is a naturally occurring flavonol with strong antiradical activity and antiproliferative effects on cancer lines, reportedly due to an impairment of cell cycle progression (51). It was also identified as an inhibitor of *T. brucei* hexokinase 1, albeit with only moderate potency (52). A closely related compound, quercertin, is toxic to *T. brucei gambiense in vitro* with an LD_50_ of 10 μM without affecting human hemopoietic cells (53). These findings, combined with myricetin's low molecular weight and considerable potency toward REL1, suggest myricetin as a possible starting point for compound optimization. These examples illustrate the potential of our assay for finding new drug leads for HAT. The unproblematic adaptation of the assay for 384-well format suggests feasibility for a 1536-well format.

The HTS described here cannot distinguish between competitive inhibitors of ATP binding (which would be expected to enter the ATP binding pocket), non-competitive inhibitors that alter REL1 activity in other ways or compounds that bind the RNA substrates. Compounds that inhibit editosome function by one of the latter two mechanisms have been described (54,55). The suramin docking studies described above and our earlier confirmation of similar compounds as inhibitors of the adenylylation step (34), strongly suggest that suramin and, by extension, NF 023, are indeed competitive inhibitors of ATP binding. The mode of inhibition of the other compounds described in the present study remains to be determined.

HAT is only one of the diseases caused by kinetoplastids (56). Chagas disease, caused by *T. cruzi* is endemic to South America and endangers tens of millions of people there (57,58). *Leishmania spp*. cause leishmaniasis which results in hundreds of thousands of deaths every year (59). All kinetoplastids have mitochondrial RNA editing ligases highly homologs to *T. brucei* REL1 (18,26). In the case of *T. brucei*, an inhibitor of the mitochondrial REL1 with potency *in vivo* is required to cross three membranes, which poses additional challenges for drug development. However, a number of important existing anti-trypanosomatid drugs have mitochondrial targets (47), a huge range of other clinically approved drugs act directly on these organelles (60), and approaches are being developed for mitochondrial specific delivery (60).

Additionally to the potential for cross-trypanosomatid drug discovery our assay also has potential as a research tool for further investigations into REL1 function. This is particularly relevant for *T. cruzi* and *Leishmania*, where genetic tools to study gene function are less developed than in *T. brucei*. *In vitro* and *in vivo* studies of REL1 biochemistry and function have traditionally relied on assays with radioactively labeled substrates (22,26,27,61). Using our new FRET-based assay drastically reduces the amount of protein and reagents needed as well as simplifying the process of activity testing.

Beyond RNA editing ligases this assay has also the potential to be used with other nucleic acid ligases. The assay format can readily be adapted to study bacteriophage ligases such as T4Rnl2 (24,62) and DNA ligases, which are pursued as potential antimicrobial and anti-cancer targets (29,63). Adaptation of the assay format to the latter enzymes would also be useful to determine if REL1 inhibitors interfere with human DNA ligase functions and thereby estimate possible side effects early during the drug development process.

In summary, the new RNA ligase activity assay presented here will aid drug discovery and basic research efforts regarding RNA editing in trypanosomes and also promises to assist the investigation of related nucleic acid processing activities in other systems.

## SUPPLEMENTARY DATA

Supplementary Data are available at NAR Online.

SUPPLEMENTARY DATA
