# Supplementary material for: A novel high-throughput activity assay for the Trypanosoma brucei editosome enzyme REL1 and other RNA ligases
Source: Nucleic Acids Res. 2015 Sep 22;44(3):e24. doi: 10.1093/nar/gkv938 (PMC4756849; doi:10.1093/nar/gkv938)
Supplement: SUPPLEMENTARY DATA [file supp_44_3_e24__index.html]

A novel high-throughput activity assay for the Trypanosoma brucei editosome enzyme REL1 and other RNA ligases — A novel high-throughput activity assay for the Trypanosoma brucei editosome enzyme REL1 and other RNA ligases — SUPPLEMENTARY DATA 

# A novel high-throughput activity assay for the *Trypanosoma brucei* editosome enzyme REL1 and other RNA ligases

## SUPPLEMENTARY DATA

- SUPPLEMENTARY DATA
