# Supplementary material for: A novel high-throughput activity assay for the Trypanosoma brucei editosome enzyme REL1 and other RNA ligases
Source: Nucleic Acids Res. 2015 Sep 22;44(3):e24. doi: 10.1093/nar/gkv938 (PMC4756849; doi:10.1093/nar/gkv938)
Supplement: SUPPLEMENTARY DATA [file supp_gkv938_nar-01943-met-g-2015-File008.pdf]

Supplementary Data

A novel high throughput activity assay for the *Trypanosoma brucei* editosome enzyme REL1 and other RNA ligases

Stephan Zimmermann[1,†,‡], Laurence Hall[1,†], Sean Riley[2], Jesper Sørensen[3], Rommie E. Amaro[3] and Achim Schnaufer[1,*]

[1]Institute of Immunology & Infection Research and Centre for Immunity, Infection & Evolution, University of Edinburgh, Edinburgh EH9 3FL, United Kingdom

[2]The Scripps Research Institute, 4122 Sorrento Valley Bl., San Diego, CA 92121, USA

[3]Department of Chemistry & Biochemistry and the National Biomedical Computation Resource, University of California, San Diego, CA 92093, USA

* To whom correspondence should be addressed. Tel: +44 (0)131 650 5548; Fax: +44 (0)131 650 6564; Email: achim.schnaufer@ed.ac.uk

[†]The authors wish it to be known that, in their opinion, the first two authors should be regarded as joint First Authors.

[‡]Deceased; this work is dedicated to the memory of Stephan Zimmermann, a wonderful colleague who is greatly missed.

Present address:

Laurence Hall, Department of Cardiovascular Sciences, University of Leicester, BHF Cardiovascular Research Centre, Glenfield General Hospital, Groby Road, Leicester LE3 9QP, United Kingdom.
Jesper Sørensen, Dart Neuroscience LLC., 12278 Scripps Summit Dr, San Diego, CA 92131, USA.

SUPPLEMENTARY METHODS

Protein docking and modelling

The Amber 99SB force field (1) was used for the proteins, parameters for suramin were developed using antechamber (2) and the general amber force field (3), charges were derived using QM calculations in Gaussian 09 (4) with the HF/6-31G* in accordance with the standard amber charge scheme (5). Dowser (6) was used to place water molecules in protein cavities and close to suramin; subsequently the system was solvated using LEaP in Amber14 (7). The system was neutralized and brought to a salt concentration of 0.1 M by adding sodium and chloride ions. The TIP4P-ew water model (8) and the monovalent ion parameters developed for this model (9) were used. The system was minimized in 4 stages slowly allowing the system to move; the first stage minimized hydrogen atoms for 2,000 steps; the second stage released water molecules and minimized for 4,000 steps; the third stage released the protein side-chains and minimized for 10,000 steps; the final stage was a 20,000

step unrestrained minimization. The system was then heated to 310 K with the protein backbone restrained for 250 ps in the NVT ensemble using a 2 fs time-step. The system was subsequently equilibrated in an NTP ensemble with 1 atm pressure in three steps of 250 ps each, slowly reducing the restraints on the protein backbone from 3 to 1 kcal/mol/Å^2. Production molecular dynamic (MD) modelling was then performed for 100 ns for each of the four models using PMEMD in Amber14 on a GPU.

SUPPLEMENTARY DISCUSSION

Further details on predicted binding mode of suramin to the REL1 active site (Fig. 6)

The sulfonate groups assume interactions similar to that of ATP, with one group interacting through a hydrogen bond with the backbone of Val88, which is bound to N7 in ATP, the second binds with a salt-bridge to the side-chain of Arg288, which connects via a water-mediated interaction to N1 in ATP. The naphthalenes described previously were only able to make subsets of these interactions due to them having only one or two of the sulfonate groups; V4 made the interaction with the side-chain of Arg288, V3 made the interaction with Val88 (10). The third sulfonate group binds to the side-chain of Arg111 and Asn92, which bind to the O2' and O3' of the ribose moiety in ATP (10). Other key protein residues involved in the binding are Glu60, Lys87, and Arg309 as detailed in Fig. 6.

Evidence that suramin can simultaneously bind to two proteins is supported by a crystal structure of *Leishmania mexicana* pyruvate kinase (*Lm*PYK) (11) together with the suramin analogue Ponceau S, although there are significant differences between their binding motifs. Firstly, the ATP binding site is inverted in *Lm*PYK, with the adenosine moiety residing close to the solvent side of the protein, while the tri-phosphate tail resides deeper in the protein. Secondly, due to the shape of the binding sites in *Lm*PYK and the protein's tetrameric state, the two Ponceau S-bound enzyme molecules come into close contact. In REL1, the ATP/suramin binding site is located on a fairly flat surface where RNA will bind, meaning the two proteins do not clash and there is room for a solvent layer in between them.

SUPPLEMENTARY REFERENCES

1. Hornak,V., Abel,R., Okur,A., Strockbine,B., Roitberg,A. and Simmerling,C. (2006) Comparison of multiple Amber force fields and development of improved protein backbone parameters. *Proteins*, **65**, 712–25.
2. Wang,J., Wang,W., Kollman,P.A. and Case,D.A. (2006) Automatic atom type and bond type perception in molecular mechanical calculations. *J. Mol. Graph. Model.*, **25**, 247–60.
3. Wang,J., Wolf,R.M., Caldwell,J.W., Kollman,P.A. and Case,D.A. (2004) Development and testing of a general amber force field. *J. Comput. Chem.*, **25**, 1157–74.
4. Frisch,M.J., Trucks,G.W., Schlegel,H.B., Scuseria,G.E., Robb,M.A., Cheeseman,J.R., Scalmani,G., Barone,V., Mennucci,B., Petersson,G.A., *et al.* (2009) Gaussian 09, Revision D.01.

5. Cornell,W.D., Cieplak,P., Bayly,C.I., Gould,I.R., Merz,K.M., Ferguson,D.M., Spellmeyer,D.C., Fox,T., Caldwell,J.W. and Kollman,P. a. (1995) A Second Generation Force Field for the Simulation of Proteins, Nucleic Acids, and Organic Molecules. *J. Am. Chem. Soc.*, **117**, 5179–5197.
6. Zhang,L. and Hermans,J. (1996) Hydrophilicity of cavities in proteins. *Proteins*, **24**, 433–8.
7. Case,D.A., Babin,V., Berryman,J.T., Betz,R.M., Cai,Q., Cerutti,D.S., Cheatham III,T.E., Darden,T.A., Duke,R.E., Gohlke,H., *et al.* (2014) AMBER 14 version.
8. Horn,H.W., Swope,W.C., Pitera,J.W., Madura,J.D., Dick,T.J., Hura,G.L. and Head-Gordon,T. (2004) Development of an improved four-site water model for biomolecular simulations: TIP4P-Ew. *J. Chem. Phys.*, **120**, 9665–78.
9. Joung,I.S. and Cheatham,T.E. (2008) Determination of alkali and halide monovalent ion parameters for use in explicitly solvated biomolecular simulations. *J. Phys. Chem. B*, **112**, 9020–41.
10. Durrant,J.D., Hall,L., Swift,R. V, Landon,M., Schnaufer,A. and Amaro,R.E. (2010) Novel naphthalene-based inhibitors of *Trypanosoma brucei* RNA editing ligase 1. *PLoS Negl. Trop. Dis.*, **4**, e803.
11. Morgan,H.P., McNae,I.W., Nowicki,M.W., Zhong,W., Michels,P. a M., Auld,D.S., Fothergill-Gilmore,L. a and Walkinshaw,M.D. (2011) The trypanocidal drug suramin and other trypan blue mimetics are inhibitors of pyruvate kinases and bind to the adenosine site. *J. Biol. Chem.*, **286**, 31232–40.
12. Gu,H. and Breaker,R.R. (2013) Production of single-stranded DNAs by self-cleavage of rolling-circle amplification products. *Biotechniques*, **54**, 337–343.

SUPPLEMENTARY FIGURE LEGENDS

Figure S1. Non-denaturing PAGE showing the RNA ligation reaction substrates and products. Lanes 1, 2 and 3 were loaded with the 16-nt 6-FAM 5’-RNA substrate, the 13-nt Cy5 3’-RNA substrate and a 29-nt dual 6-FAM/Cy5-labelled DNA oligo (see legend to Supplementary Figure S3D for sequence), respectively. Lane 4 was left empty. Lanes 5 to 8 show results of ligation reactions after denaturation by heat and in the presence of competitor DNA, while lanes 9 to 12 show reactions that had been loaded without prior denaturation. Reactions were carried out in the presence of 0.2 µg or 2.0 µg REL1, as indicated. Mock reactions in the absence of REL1 (lanes 7 and 11) or in the absence of gRNA (lanes 8 and 12) were included as controls. The top, middle and bottom panels show the signals from the 5’-RNA substrate (6-FAM channel), 3’-RNA substrate (Cy5 channel) and FRET (6-FAM laser, Cy5 filter for reading), respectively. The gel analysis confirmed the following:

Lane 1: The 5’-RNA showed a strong signal in the 6-FAM channel but weak to no signals in the Cy5 and FRET channels.

Lane 2: The 3’-RNA showed a strong signal in the Cy5 channel but weak to no signals in the 6-FAM and FRET channels.

Lane 3: The dual 6-FAM/Cy5-labelled DNA oligo showed strong signals in all three channels.

Lanes 5 to 8: Incubation of 5'-RNA and 3'-RNAs with gRNA and REL1 resulted in the formation of two main species of higher molecular weight that exhibited signals in all three channels. The lower band migrated slightly above the 29-nt dual-labelled ssDNA in lane 3; its length is consistent with it being the single-stranded 5'-RNA + 3'-RNA ligation product (note that ssRNA typically shows a slower mobility on non-denaturing PAGE gels than ssDNA of the same length (12)). The migration of the upper band is consistent with it being the double-stranded duplex of 5'-RNA + 3'-RNA ligation product and gRNA. Apparently some of the duplex was either not completely disrupted by heat denaturation, or it re-annealed partially after loading onto the gel. Crucially, however, the presence of both bands was fully dependent on both REL1 (lane 7) and gRNA (lane 8): omitting either REL1 or gRNA resulted in a very low signal in the FRET channel caused by the free 5'-RNA and 3'-RNA.

Lanes 9 to 12: Without denaturation, as expected, the presence of the double-stranded duplex of 5'-RNA, 3'-RNA and gRNA was independent of the addition of REL1 (compare lane 11 with lanes 9 and 10). In the absence of gRNA, only the input 5'-RNA and 3'-RNA species were observed (lane 12).

Figure S2. Analysis of the RNA ligation products by denaturing PAGE (7 M urea). Incubation of the annealed 5'-RNA + 3'-RNA + gRNA duplex with REL1 results in formation of a larger ligation product that gives an efficient FRET signal (bottom panel, lanes 1 and 2; more REL1 results in more product). Product formation is fully dependent on REL1 (lane 3 = no REL1) and gRNA (lane 4 = no gRNA).

Figure S3. Optimization of assay conditions. (**A, B**) Evaluation of 6-FAM vs. Cy3 as donor fluorophore. (A) 5'-RNA labeled with 6-FAM and 3'-RNA labeled with Cy5 (attachment sites as depicted in Fig. 1B) were annealed to gRNA as described in Materials and Methods under 'Initial RNA annealing and ligation conditions'; control reactions without gRNA were carried out in parallel as indicated. The ligation step was omitted. Means and standard deviations for three replicates are shown. Stop buffer was added and samples were denatured or not at 96°C before reading, as indicated. (B) As described for (A), but with Cy3 as donor fluorophore. (**C**) FRET signal of assays carried out with HEPES ligation buffers of the pH values indicated. Otherwise the assay was carried out as described in Materials and Methods under 'Initial RNA annealing and ligation conditions'. For each pH value, means and standard deviations for six replicates are shown. (**D**) Representative standard curve obtained by measuring fluorescence for defined concentrations of DNA oligo 5-- AAGTATGAGACG/T-6FAM/AGGAT/T-Cy5/GGAGTTATAG-3'. Means and standard deviations for three replicates are shown. Note that this sequence corresponds to the ligated product shown in Fig. 1B. For relevant assays, a separate standard curve was established using the exact same spectrofluorometer gain settings as used in that assay. (**E**) Adding fresh ATP after 20 min restarted the reaction. Reactions were carried out as described in Materials and Methods under 'Initial RNA annealing and ligation conditions', except [ATP] was as indicated. For the '2 + 2' reaction, fresh ATP (at a final concentration of 2 μM) was added after 20 min. Means and standard deviations for three replicates are shown.

Figure S4. Non-denaturing PAGE showing inhibition of RNA ligation reaction by compound NSC42067. Lanes 1, 2 and 3 were loaded with the 16-nt 6-FAM 5'-RNA substrate, the 13-nt Cy5 3'-RNA substrate and a 29-nt dual 6-FAM/Cy5-labelled DNA oligo, respectively. Lane 4 was left empty. Lanes 5 to 8 show products from standard reaction conditions and controls. Lanes 9 to 11 show the effects of increasing concentrations of NSC42067 in the assay. Ligation reactions were carried out under optimized conditions. The gel analysis confirmed the following:

Lanes 1 - 3: See Supplementary Figure S1.

Lanes 5 to 7: As before (compare Supplementary Figure S1), incubation of 5'- and 3'-RNAs with gRNA and REL1 resulted in the formation of two main species of higher molecular weight that exhibited signals in all three channels (lanes 6 and 7). The intensity of both bands increased with longer incubation time (compare lane 6 = 5 min with lane 7 = 30 min) and was dependent on the presence of REL1 (lane 5).

Lanes 8 to 10: Increasing concentrations of NSC42067 inhibit formation of the ligation product.

Figure S5. Structures of the hit compounds selected for validation.

Figure S6. Scatter plot showing the results of the second pilot screen. Compounds of the Maybridge library were incubated at 10 µM concentration in 384-well plates with REL1 (7.8 nM) in the presence of ATP (10 µM) and RNA substrate (0.5 µM). Stop buffer was added after 30 min. Wells containing no REL1 were used as 'low control' (LC, red), wells containing no compounds as 'high control' (HC, blue) and compound wells (CMPND, green) were normalized to % inhibition using high control and low control.

Figure S7. Plot showing the root-mean square deviation (RMSD) of the four generated REL1-suramin models as a function of simulation-time based on the backbone carbon alpha atoms in the first (**A**) and second (**B**) enzyme.

heat denatured (lanes 5–8) | not denatured (lanes 9–12)

lane	1	2	3	4	5	6	7	8	9	10	11	12

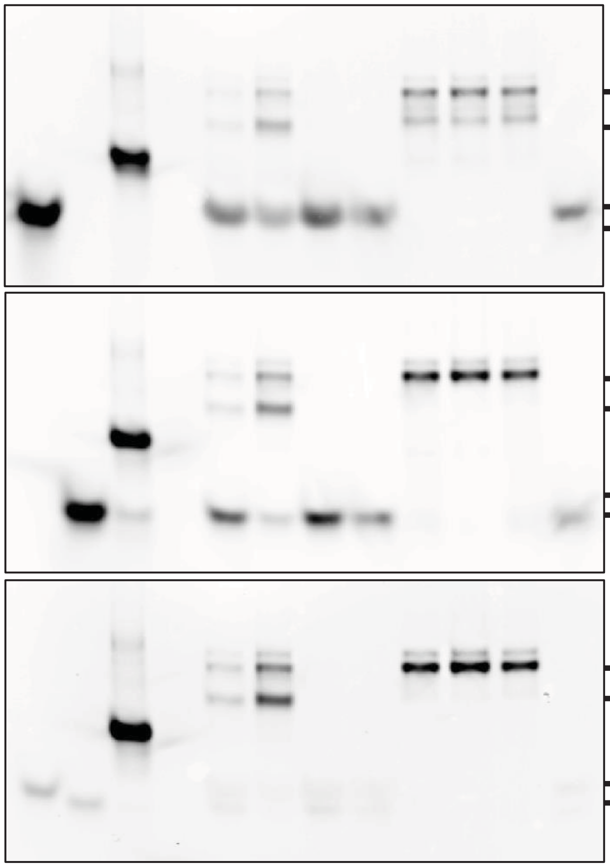

lane	1	2	3	4	5	6	7	8	9	10	11	12
6-FAM 5’-RNA	x				x	x	x	x	x	x	x	x
Cy5 3’-RNA		x			x	x	x	x	x	x	x	x
gRNA					x	x	x		x	x	x	
REL1 (µg)					0.2	2.0		0.2	0.2	2.0		0.2
6-FAM/Cy5 oligo			x									

Figure S1

lane	1	2	3	4

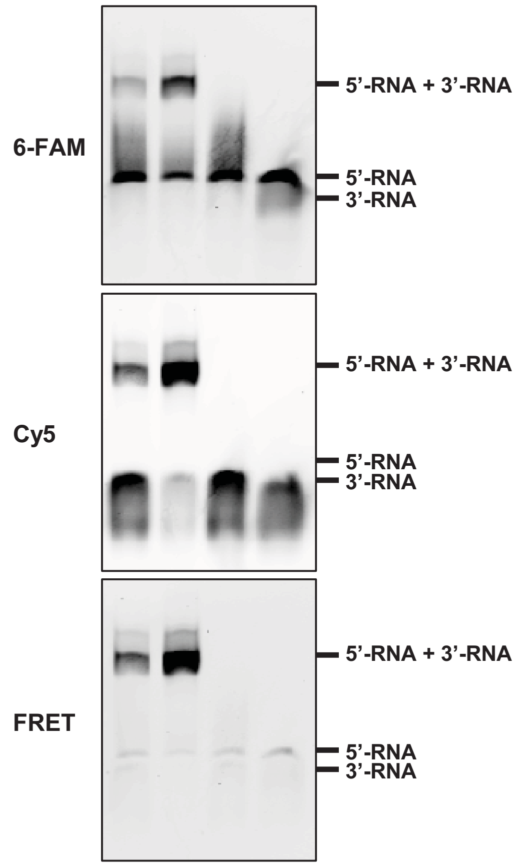

lane	1	2	3	4
6-FAM 5'-RNA	x	x	x	x
Cy5 3'-RNA	x	x	x	x
gRNA	x	x	x	
REL1 (µg)	0.2	2.0		2.0

Figure S2

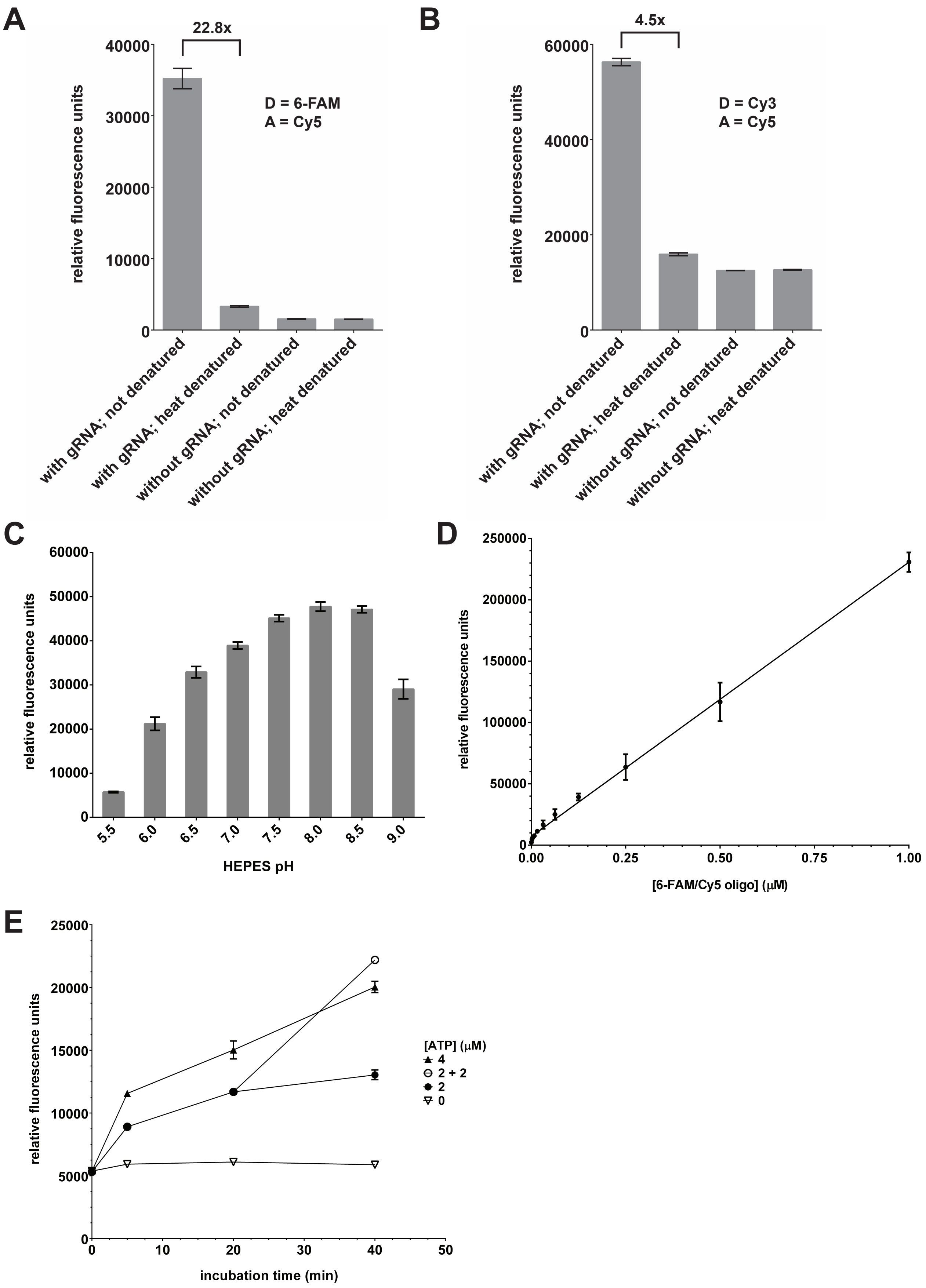
A
22.8x
40000
30000
20000
10000
0
relative fluorescence units
D = 6-FAM
A = Cy5
with gRNA; not denatured
with gRNA; heat denatured
without gRNA; not denatured
without gRNA; heat denatured
B
4.5x
60000
40000
20000
0
relative fluorescence units
D = Cy3
A = Cy5
with gRNA; not denatured
with gRNA; heat denatured
without gRNA; not denatured
without gRNA; heat denatured
C
60000
50000
40000
30000
20000
10000
0
relative fluorescence units
5.5
6.0
6.5
7.0
7.5
8.0
8.5
9.0
HEPES pH
D
250000
200000
150000
100000
50000
0
relative fluorescence units
0.00
0.25
0.50
0.75
1.00
[6-FAM/Cy5 oligo] (μM)
E
25000
20000
15000
10000
5000
0
relative fluorescence units
0
10
20
30
40
50
incubation time (min)
[ATP] (μM)
4
2 + 2
2
0

Figure S3

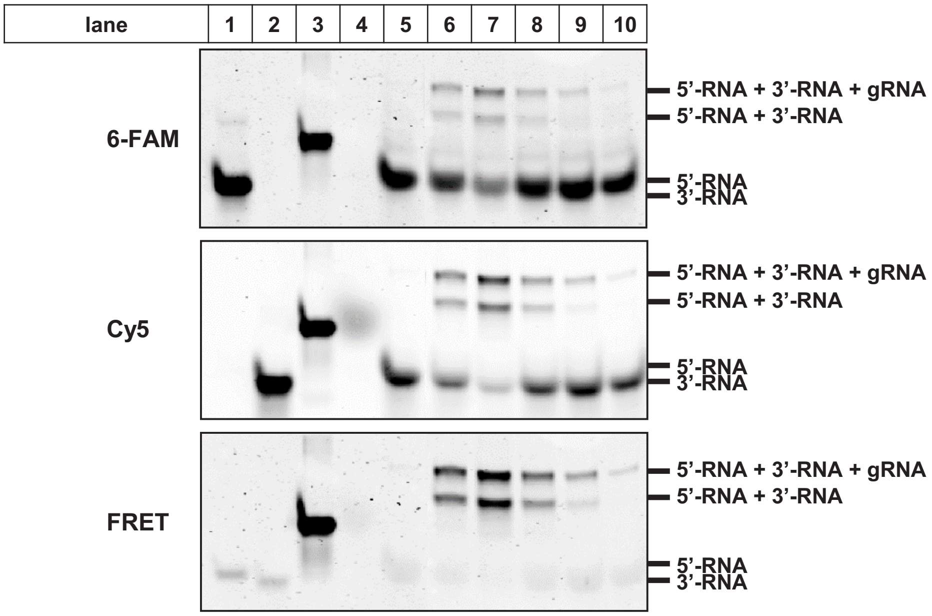

lane	1	2	3	4	5	6	7	8	9	10
6-FAM 5’-RNA	x				x	x	x	x	x	x
Cy5 3’-RNA		x			x	x	x	x	x	x
gRNA					x	x	x	x	x	x
REL1						x	x	x	x	x
[NSC42067] (μM)					0	0	0	5	10	50
ligation time (min)					30	5	30	30	30	30
6-FAM/Cy5 oligo			x							

Figure S4

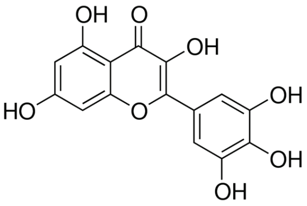

Myricetin

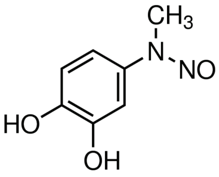

Methyl-3,4-dephostatin

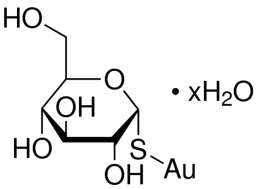

Aurothioglucose hydrate

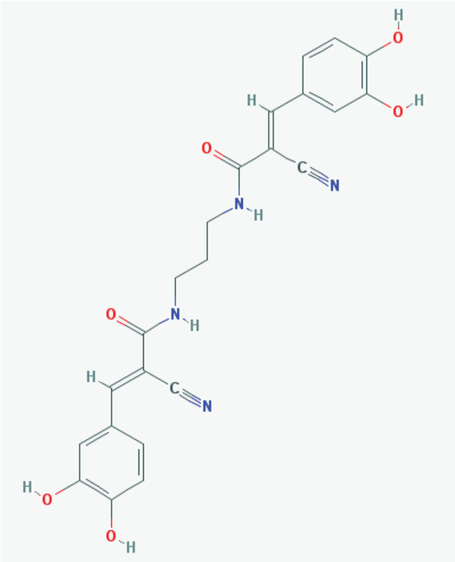

Tyrphostin AG 537

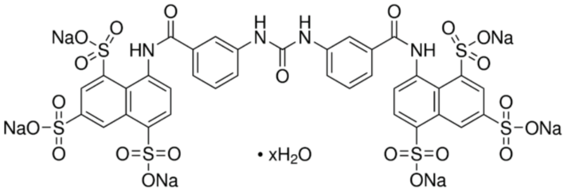

NF 023 hydrate

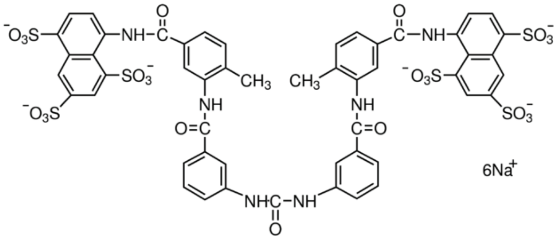

Suramin

Figure S5

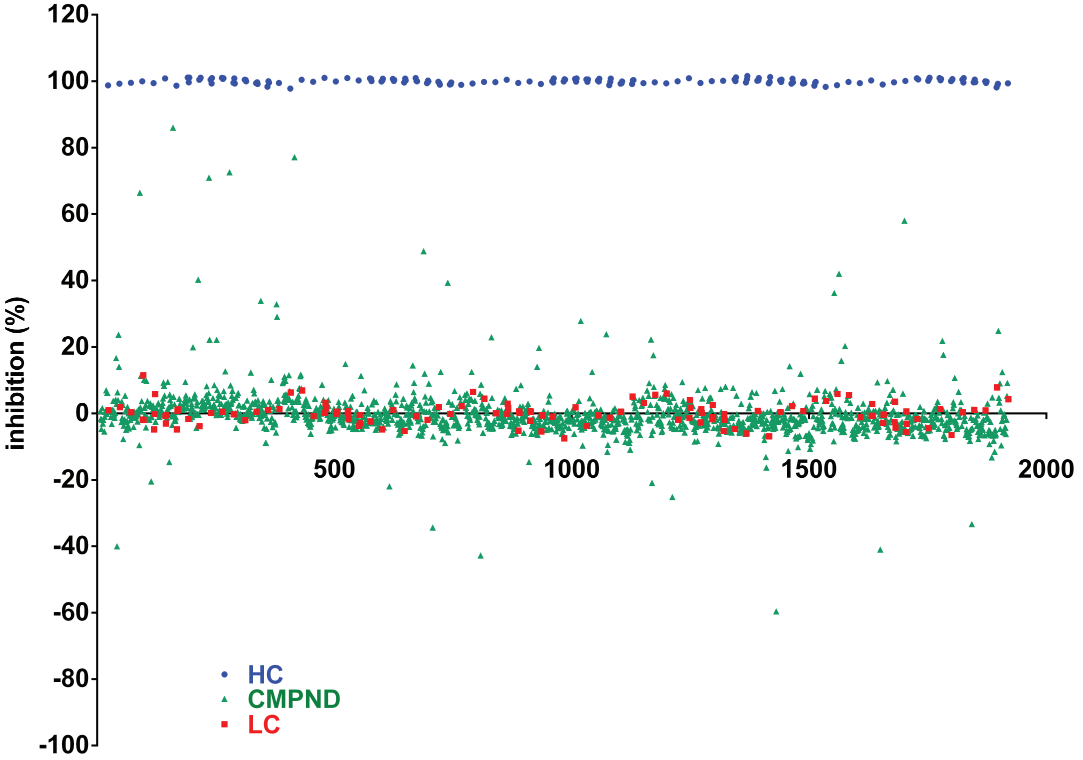

120
100
80
60
40
20
0
-20
-40
-60
-80
-100
inhibition (%)
500
1000
1500
2000
HC
CMPND
LC

Figure S6

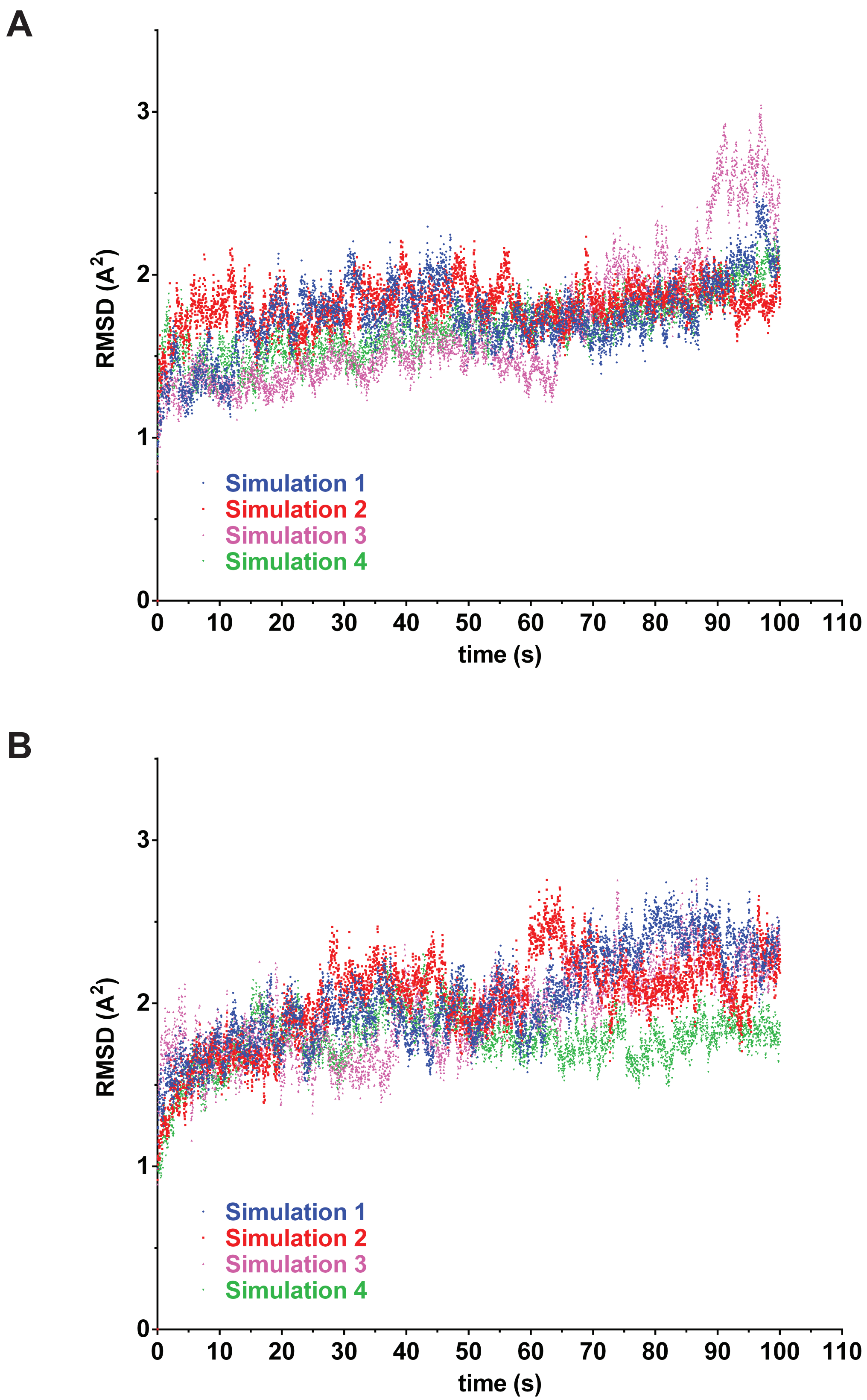
A
RMSD (A2)
3
2
1
0
0 10 20 30 40 50 60 70 80 90 100 110
time (s)
Simulation 1
Simulation 2
Simulation 3
Simulation 4
B
RMSD (A2)
3
2
1
0
0 10 20 30 40 50 60 70 80 90 100 110
time (s)
Simulation 1
Simulation 2
Simulation 3
Simulation 4

Figure S7
